# Comparative evaluation of ultrasonography with clinical respiratory score in diagnosis and prognosis of respiratory diseases in weaned dairy buffalo and cattle calves

**DOI:** 10.1186/s40781-018-0187-3

**Published:** 2018-12-03

**Authors:** Hussein Awad Hussein, Cagri Binici, Rudolf Staufenbiel

**Affiliations:** 1 Internal Veterinary Medicine, Department of Animal Medicine, Faculty of Veterinary Medicine, Assiut University, Assiut, 71526 Egypt; 2 Klinik für Klauentiere, Freie Universität Berlin, 14163 Berlin, Germany

**Keywords:** Bronchopneumonia, Calves, Emphysema, Interstitial pulmonary syndrome, Prognosis

## Abstract

**Background:**

Respiratory troubles have economic impacts in countries where livestock industry is an important segment of the agricultural sector, as well as these problems may cause significant economic losses for bovine producers. Various practical methods are used to assess diseases that affect the bovine respiratory system. Ultrasonography is a noninvasive tool that has been used frequently in diagnosis of various animal diseases. The present study was designed to establish whether thoracic ultrasonography is a diagnostic tool for detection of respiratory troubles in weaned buffalo and cattle calves, as well as to assess its prognostic value in comparison with clinical respiratory scores. Thirty five (15 buffalo and 20 cattle) calves were included. Twelve (6 buffalo and 6 cattle) clinically healthy calves were enrolled as controls.

**Results:**

Based on physical examinations, clinical respiratory scores (CRS), ultrasound lung scores (ULS) and postmortem findings, animals were classified into 4 groups as pulmonary emphysema (*n* = 8), interstitial pulmonary syndrome (*n* = 7), bronchopneumonia (*n* = 12), and pleurisy (*n* = 8). The mean values of CRS and ULS were significantly higher in diseased calves (*P* < 0.01). In calves with pulmonary emphysema and interstitial syndrome, thoracic ultrasonography revealed numerous comet-tail artifacts, which varied in numbers and imaging features. Furthermore, variable degrees of pulmonary consolidation with alveolograms and bronchograms were noticed in bronchopneumonic calves. In addition, thick irregular or fragmented pleura with pleural effusions and fibrin shreds were imaged in calves with pleurisy. A weak correlation was calculated between CRS and ULS (*r* = 0.55, *P* < 0.01). Hematologically, the counts of white blood cells, activities of aspartate aminotransferase and partial tensions of carbon dioxide were significantly increased in all diseased groups. Serum concentrations of total globulins were higher in claves with bronchopneumonia (*P* < 0.05). The partial tension of oxygen was decreased in all diseased calves (*P* < 0.05).

**Conclusions:**

Thoracic ultrasonography is a diagnostic tool for various lung troubles and assessment the grade and severity of pulmonary diseases, as well as it can be used as a follow-up tool for evaluating the prognosis of respiratory troubles and monitoring the efficacy of therapies.

## Background

Bovine respiratory disease and diarrhea are the most common diseases affecting the health of dairy calves [1]. Bovine respiratory diseases are multifactorial, being caused by various viruses, bacteria, and mycoplasma and are dependent on predisposing factors as well as management errors [2].

Respiratory troubles have economic impacts in countries where livestock industry is an important segment of the agricultural sector, as well as these problems may cause significant economic losses for bovine producers. Respiratory diseases cause an estimated $800 million to $900 million annually in economic losses from death, reduced feed efficiency, and treatment costs [3, 4]. In dairy calves, respiratory diseases were associated with a lower body weight [5], reduced average daily gain [6] and a potential for increased mortality [7]. In beef calves, medical costs attributable to the treatment of respiratory diseases are substantial, and the economic impacts of respiratory problems on carcass merit and meat quality further increase the economic costs [8].

Various practical methods are used to assess diseases that affect the bovine respiratory system, including auscultation, percussion, blood ancillary tests, radiography, ultrasonography, and more invasive procedures such as pulmonary aspirations and biopsies [9]. Ultrasonography is a noninvasive tool that has been used frequently in diagnosis of cardiac diseases in buffalo [10]. Furthermore, ultrasonography is a good choice for imaging and describing suppurative pneumonia in cattle [11].

From the clinical point of view, it is of great interest to make a precise diagnosis, as it permits rapid evaluation and decision making regarding treatment options and avoids wasteful supportive treatments. In comparison with clinical respiratory score, therefore, thoracic ultrasonography was used to assign the spread and evaluate the prognostic aspects of respiratory diseases in weaned dairy buffalo and cattle calves. The results were compared with the clinical and postmortem findings.

## Methods

### Animals, history and clinical examination

A total of 35 calves (15 buffalo and 20 cattle) aged between 4 and 7 months were included in the present study. Fifteen cattle calves were examined at the Clinic of Ruminant and Swine, Faculty of Veterinary Medicine, Free University of Berlin, Germany. The remaining 20 calves (15 buffalo and 5 cattle) were examined at the Department of Animal Medicine, Faculty of Veterinary Medicine, Assiut University, Egypt. Based on the owners’ complaints, all animals were admitted because of inappetance, nasal discharges, coughing, and variable degrees of dyspnea. There was no history of previous medications either by the farmers or field veterinarians in any of the calves examined. All animals were examined clinically as described previously [12], which included general heath condition and auscultation of the heart and lungs. Furthermore, rectal temperature and respiratory and heart rates were also recorded. After complete and thorough clinical examinations, 12 calves (6 buffalo and 6 cattle) were included in this study as controls. These animals did not have a history of previous respiratory illness and their physical examination was within normal ranges.

### Clinical respiratory score (CRS)

Each calf was assessed using respiratory scoring chart from the University of Wisconsin [1]. This scoring system based on assessment of five clinical signs including rectal temperature, cough, eye and nasal discharge, and ear position. Information on nasal discharge, ocular discharge, ear and head position, rectal temperature, and the frequency of induced or spontaneous coughing were recorded for each calf. Each clinical sign partitioned into four levels of severity (from 0 to 3) as 0 indicates the lowest risk of being sick and 3 with the highest risk of respiratory disease. It is recommended to treat calves because of high respiratory disease if the CRS is ≥5, and to observe calves with scores of 4. Calves with ≤3 are considered clinically healthy.

### Thoracic ultrasonography

Ultrasonographic examination of the pleura and lungs was carried out as described previously [13]. In preparation, the area from 3rd to 12th intercostal spaces (ICS) was clipped, shaved, and scrubbed with alcohol to remove excess oil, and coupling gel was applied. Thoracic ultrasonography was carried out using 4–6 MHz micro convex ultrasound transducer (MyLab™One VET, Esaote, Netherlands), where the frequency was changed according to the examination’s requirement. In both sides, each lung was examined dorsoventrally with the transducer held parallel to the ribs. The lung was considered normal if the characteristic features of well-ventilated lung tissue with a smooth visceral surface was seen and if a pleural reflective band and reverberation artifacts were observed [13]. The different abnormalities noted were the presence of comet-tail artifacts, pleural fluid accumulation, pleural irregularity and thickening, and consolidated lung. Comet-tails were observed from the pleura to the deeper part of the ultrasonogram during the scanning of the site [14]. Pleural effusion was diagnosed by accumulation of fluid between the parietal and visceral layers of pleura. Pleural irregularity and thickening was noted if, in contrast to a normal thin smooth hyperechoic line, the pleural line was serrated with an irregular shape and the pleural line thickness higher than 1 mm [7]. Pulmonary consolidation was noted if the lung tissue appeared hypoechoic and its echo texture looked like liver parenchyma. Based on the degree of lung consolidation and the number of lobes involved, ultrasonographic lung score (ULS) (0–5) was used to categorize calves as described previously [7]. Bronchoaerograms was defined as hyperechoic spots indicated small bronchi filled with air.

### Blood sampling, hematological and biochemical analyses

Two venous blood samples were collected from jugular vein; one placed in tubes containing ethylene diamine tetraacetic acid (EDTA) as anticoagulant and the other in plain tubes. The haematocrit, haemoglobin concentration, total red blood cell count (RBCs) and total white blood cells count (WBCs) were measured in the samples containing EDTA using veterinary automated cell counter. After centrifugation of the second blood sample, serum samples were collected and then frozen at − 20 °C till biochemical analysis. In the serum samples, commercial test kits were used to determine the concentrations of total proteins and albumin. The activities of aspartate aminotransferase (AST) and γ-glutamyl transpeptidase (GGT) were also measured in serum samples. The biochemical analyses of the parameters were spectrophotometrically measured according to the standard protocols of the test kits using UV spectrophotometers. From each animal, a third blood sample was collected from the caudal auricular artery and placed in a syringe contains heparin as anticoagulant for arterial blood gas analysis. Arterial blood gas indices were estimated using blood gas analyser (ABL 5, Radiometer, Copenhagen, Denmark).

### Postmortem examination

Necropsies were performed on 15 calves (7 buffalo and 8 cattle). Buffalo calves died 1 day after beginning of therapy, while the cattle calves euthanased for welfare reasons.

### Statistical analyses

The data were statistically analyzed using SPSS (SPSS analytical program for Windows Version 20; SPSS GmbH, Munich, Germany). The normality of all variables was tested using Kolmogorov-Smirnov test. All variables were normally distributed. Differences between diseased and control animals were assessed by one-way analysis of variance (ANOVA). Pearson’s correlation coefficient (*r*) was used to determine the relationship between parameters. Post hoc Bonferroni multiple comparison test was used to assess the possible variation in variables with the preceding of therapy. For all statistical examinations, results were considered significant at *P* < 0.05. All data are listed as mean ± SD.

## Results

Based on clinical examination including, coughing, tachypnoea, inspiratory dyspnea, nasal discharges, abnormal lung sounds and CRS as well as ultrasonographic findings and postmortem findings for dead and euthanized cases, diseased calves were classified into 4 groups: pulmonary emphysema (*n* = 8), interstitial pulmonary syndrome (*n* = 7), bronchopneumonia (*n* = 12), and pleurisy (*n* = 8).

### Clinical presentation and respiratory score

**Fig. 1:**
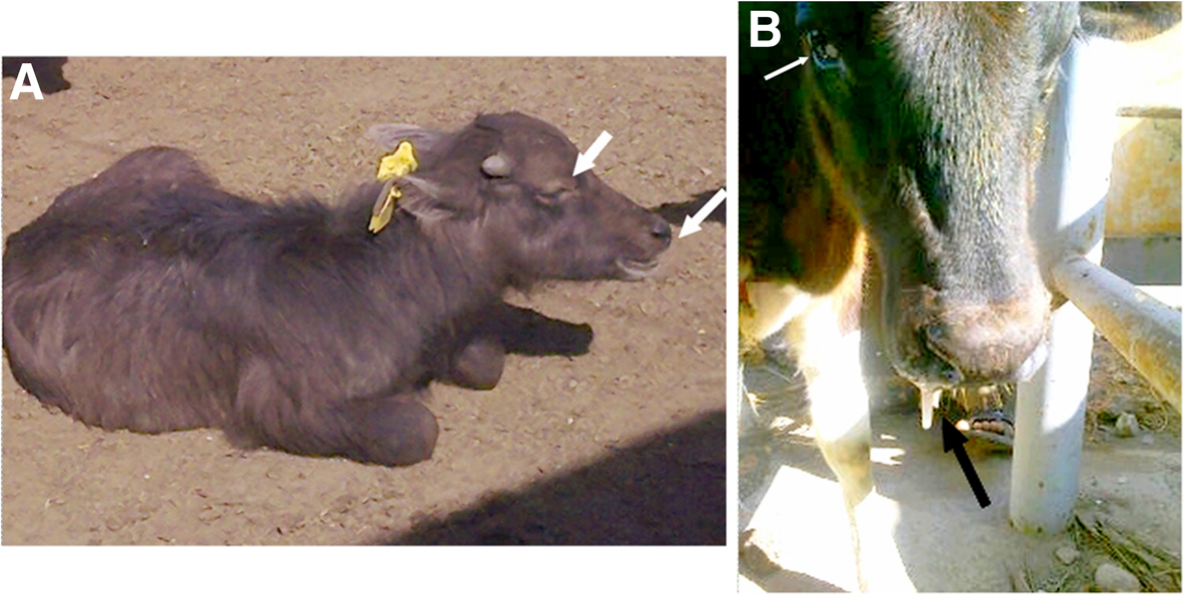
Clinical presentations of calves with respiratory troubles. **a** Dullness, lacrimation and mouth breathing are signs in a buffalo calf with interstitial pulmonary syndrome (CRS = 5); **b** ocular secretion (white arrow) and purulent nasal discharges (black arrow) in a cattle calf with bronchopneumonia (CRS = 6). CRS, Clinical respiratory score

Clinical presentations were varied according to the respiratory disease condition. At admission, the general condition of most calves was deteriorated. The most observable clinical presentations were dullness, difficult inspiration (mouth breathing, widening of nostrils) (Fig. 1a), and variable degrees of nasal discharges, which was either unilateral or bilateral, that ranged from mucopurulent to purulent in consistency (Fig. 1b). The values of rectal temperature ranged from 38.7 °C to 41 °C. The lowest and highest values of body temperature were noticed in calves with emphysema and pleurisy, respectively. In addition, 8 calves with bronchopneumonia showed pyrexia (mean value of rectal temperature was 40 ± 0.6 °C). The mean value of pulse rate was 92beats/min (range, 78–110). The mean respiratory rate was 43breaths/min (range, 28–52). Upon percussion of the lungs, 5 cases with pulmonary emphysema showed increased and 4 cases with bronchopneumonia had reduced resonance. Upon auscultation of lungs, 3 cases with interstitial pulmonary syndrome exhibited exaggerated vesicular sounds, 5 cases with bronchopneumonia showed moist ráles and 2 cases with pleurisy had pleuritic frictional sounds, resembling two sheets of sandpaper rubbing against each other. Four cases with pulmonary emphysema showed reduced vesicular breath sounds. In contrast, six cases (2 with interstitial pulmonary syndrome and 4 with bronchopneumonia) exhibited no lung sounds by auscultation. The mean values of CRS were significantly higher in all diseased groups in comparison with their corresponding control ones (*P* < 0.01) (Table 1).

**Table 1:** Clinical respiratory and ultrasound lung scores in calves with respiratory troubles (*n* = 35)

	Buffalo calves			Cattle calves		
	Control (*n* = 6)	Emphysema (*n* = 8)	Interstitial pulmonary syndrome (*n* = 7)	Control (*n* = 6)	Bronchopneumonia (*n* = 12)	Pleurisy (*n* = 8)
CRS	1.5 ± 0.3	4.3 ± 0.3^**^	5.1 ± 0.5^**^	1.7 ± 0.2	5.2 ± 0.2^**^	5 ± 0.4^**^
ULS	0.0 ± 0.0	2.3 ± 0.3^**^	2.1 ± 0.3^*^	0.0 ± 0.0	3.1 ± 0.2^**^	3.4 ± 0.3^**^

*CRS* Clinical respiratory score, *ULS* ultrasound lung score

^*^or ^**^ Means within a row of the same species are significantly different (*P* < 0.05) or (*P* < 0.01), respectively from values of the corresponding control animals

### Ultrasonographic and postmortem findings

**Fig. 2:**
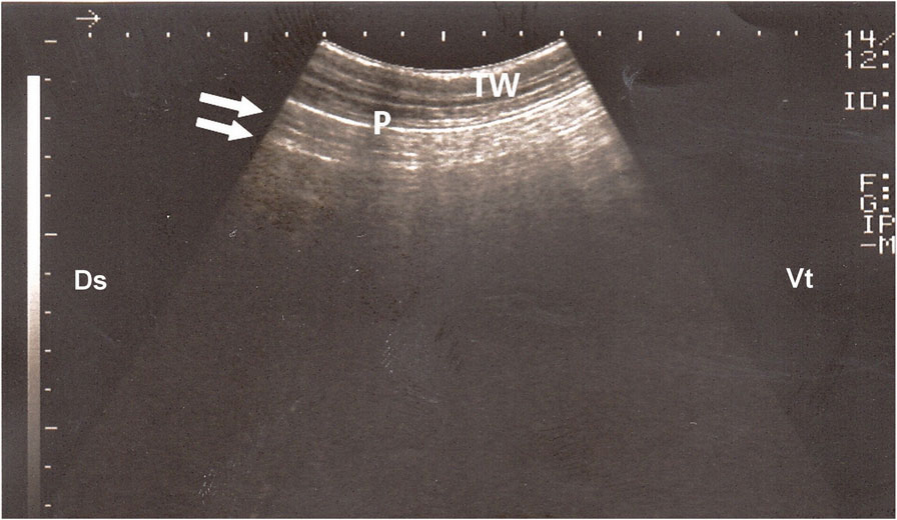
Ultrasonogram of a normal lung generated using a 5 MHz sector probe: hyperechoic pleural line (P) with less echogenic lines parallel to the pleural surface (arrows) represent reverberation artifacts. TW, Thoracic wall; P, Pleura; Ds, Dorsal; Vt, Ventral

The ultrasonographic appearance of normal aerated lung of control groups was characterized by the uppermost white linear echo, representing parietal and visceral pleurae, with reverberation artifacts (Fig. 2). During respiration, the visceral pleura was seen moving with a real-time examination. Neither lung consolidation nor pleural effusions were visualized in these normal calves. The postmortem examinations of these calves confirmed these ultrasonographic results.

**Fig. 3:**
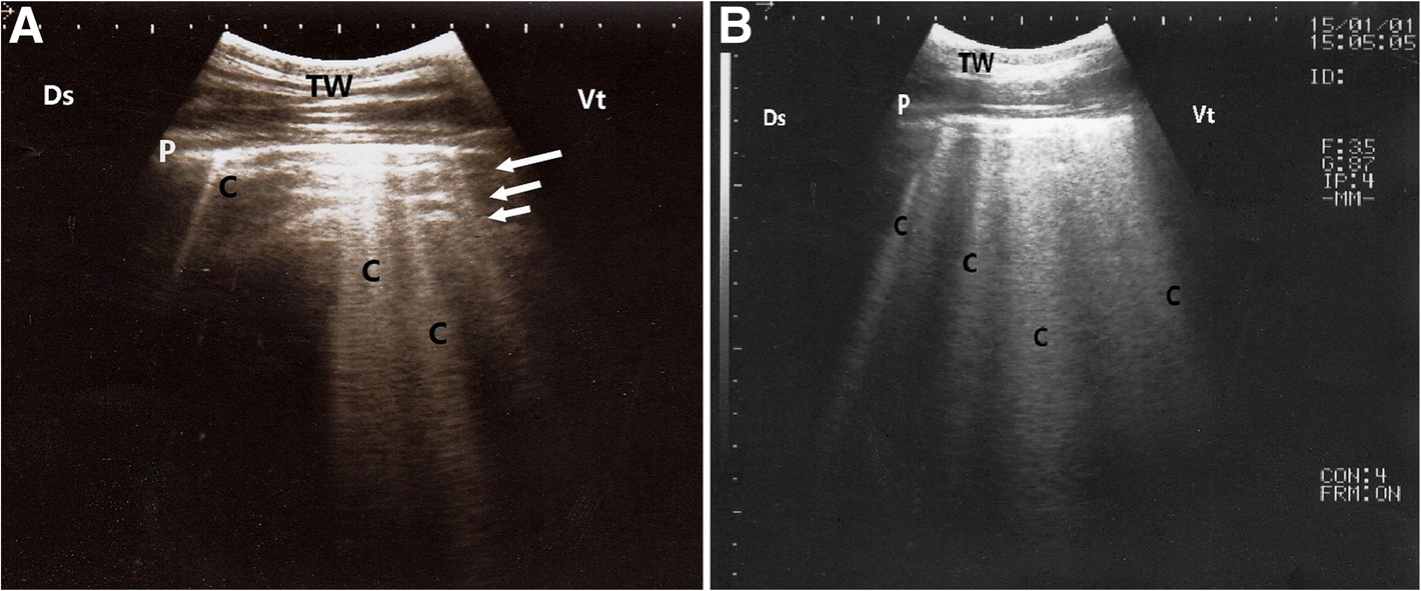
Ultrasonograms of the lung of buffalo calves with pulmonary emphysema. **a** A mild case of pulmonary emphysema shows few echogenic bands (C) run from the lung surface with reverberation artifacts (arrows); **b** A severe case of pulmonary emphysema shows multiple echogenic bands (C) originate from the pleural surface. TW, Thoracic wall; P, Pleura; Ds, Dorsal; Vt, Ventral; C, Comet-tail artifacts

**Fig. 4:**
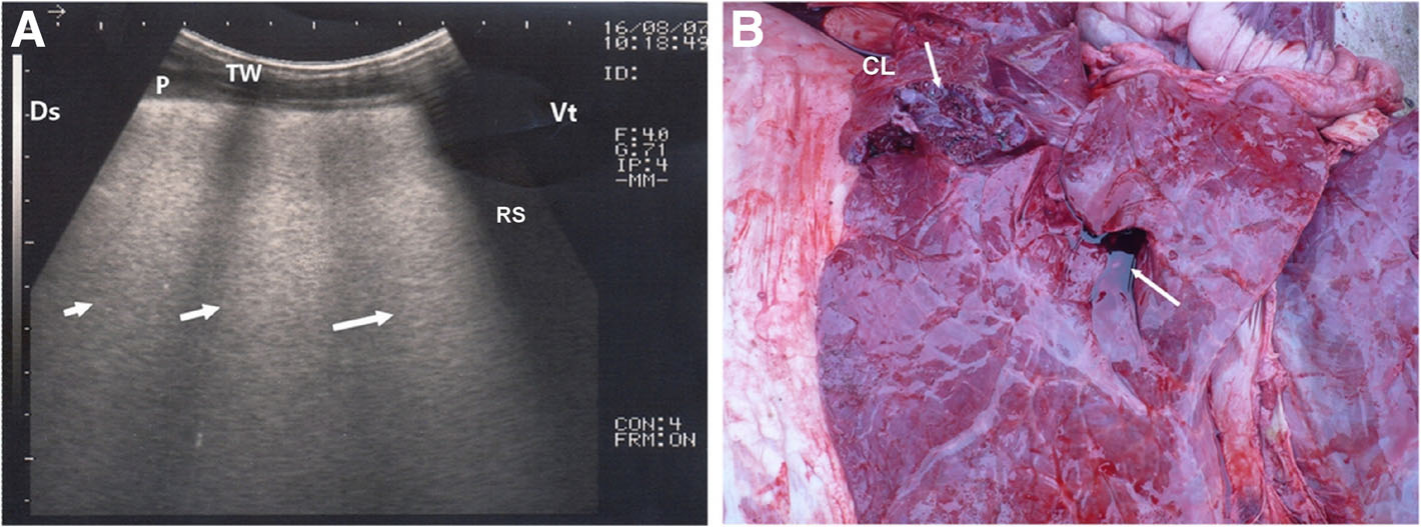
Ultrasonogram and postmortem finding of a buffalo calf with interstitial pulmonary syndrome. Image (**a**) shows multiple ultrasound lung comets fanning out from the pleural line to the edge of the image (arrows), white lung. Image (**b)** represents postmortem findings of the same calf that shows severely congested and edematous lung. TW, Thoracic wall; P, Pleura; Ds, Dorsal; Vt, Ventral; RS, Rib shadowing

In calves with pulmonary emphysema, the ultrasonographic examination of chest showed numerous comet-tail artifacts in the form of bright echogenic bands extending at the lung surface and running perpendicular to the pleura. These comet-tail artifacts ranged from 3 (Fig. 3a) to 4 (Fig. 3b) in number. The most affected lung lobes were the intermediate (5 calves), then the cranial (3 calves) lobes. The Thoracic ultrasonography of buffalo calves with interstitial pulmonary syndrome revealed presence of multiple ultrasound lung comets fanning out from the pleural line to the edge of the image. These lung comets were broader than that in emphysema and the entire ultrasound image appeared white in colour (Fig. 4a). At postmortem examinations of these calves, their lungs were severely congested and edematous (Fig. 4 b).

**Fig. 5:**
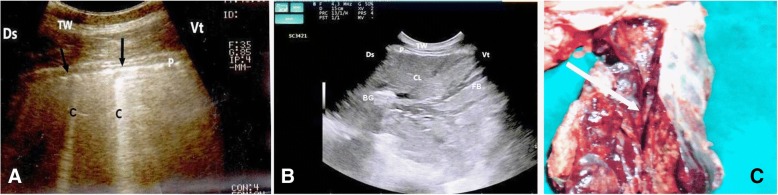
Ultrasonograms of cattle calves with bronchopneumonia. **a** An image for a mild case shows a small hypoechoic zone on the surface of the lung (arrow, diameter: 7 mm), representing fluid alveologram with comet-tail artifact (C). **b** An image for the cranial lobe of a severely bronchopneumonic calf shows consolidated lung with ramified fluid bronchograms (FG). **c** This image represents postmortem findings of the same case in image B showing consolidation of the cranial lobe (arrow). TW, Thoracic wall; P, Pleura; Ds, Dorsal; Vt, Ventral; CL, Consolidated lung

**Fig. 6:**
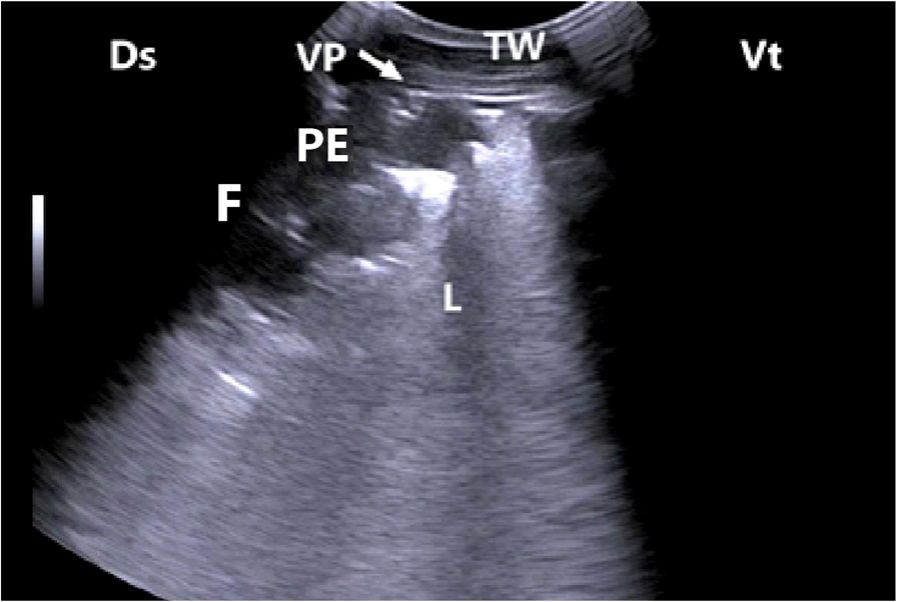
Ultrasonogram of a lung of cattle calf with fibrinous pleurisy exhibits interrupted visceral pleura (VP) with accumulation of fibrin (F) and inflammatory exudates forming pleural effusion (PE). TW, Thoracic wall; Ds, Dorsal; Vt, Ventral; L, Lung

In calves with bronchopneumonia, the ultrasonographic appearance of diseased lungs varied depending on the degree and type of lesions. Four cases showed small hypoechoic circular zones (about 7 mm in diameter) at the lung surface, representing a superficial fluid alveologram with a comet-tail artifact (Fig. 5a). The ultrasonographic features of the lung tissue in ten calves with bronchopneumonia appeared hypoechoic and its echo texture may look like liver parenchyma, representing pulmonary consolidation and containing ramified fluid bronchgrams (Fig. 5b). These consolidated areas have a homogenous and echoic basic texture with well defined borders. Lesions of lung consolidation were found on the left side of the thorax in 7 cases and on the right side of the thorax in 3 cases. In three cases, air-filled alveoli were imaged in this hepatized lung tissue, appearing as hyperechoic spots in the consolidated parenchyma. Occasionally, hyperechoic reflective bands of an air-filled bronchus were detected in the pulmonary tissue, representing air bronchoaerograms. The most affected lobes were the cranial (8 calves) (Fig. 5c), then the cardiac (2 calves) and intermediate (2 calves) lobes. Thoracic ultrasonographic examination of calves with pleurisy revealed presence of thick fragmented visceral pleura and filling of pleural sac with echogenic fibrin shreds and anechoic exudates (Fig. 6). Moderate pleural effusion was seen in 3 cases, while pleural irregularity was observed in 4 of 8 cattle calves with pleurisy. One case showed roughened texture of the pleural surface causing narrow streaks of comet tails.

**Fig. 7:**
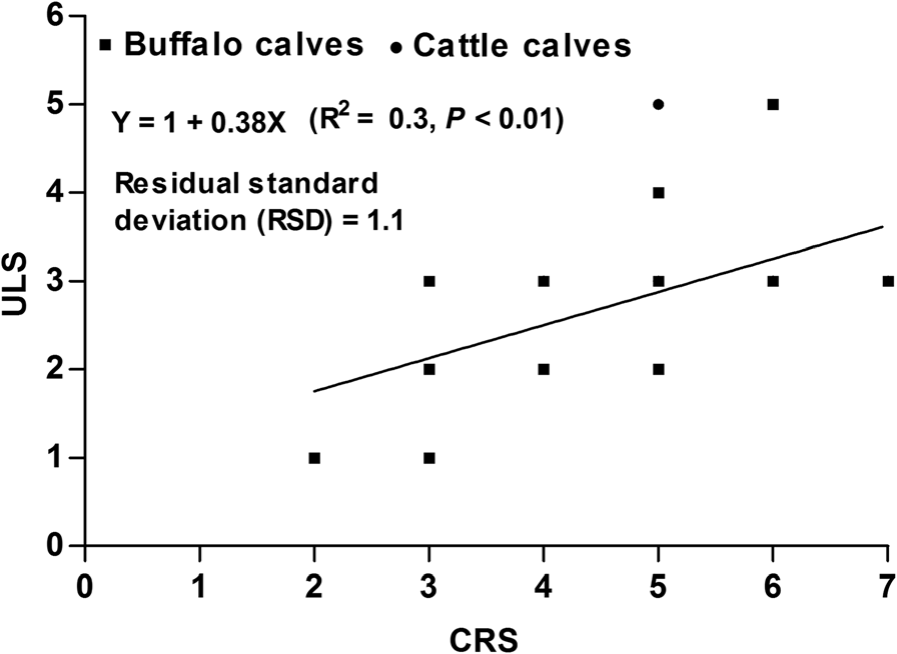
A scatter plot for the relationship between the clinical respiratory scores (CRS) and ultrasound lung scores (ULS) in calves with respiratory troubles

Pearson’s correlation coefficients were calculated to find the relationship between ULS and CRS. A weak correlation was noticed between the two variables (*r* = 0.55, *P* < 0.01) (Fig. 7).

### Hematological and biochemical findings

Table 2 summarizes the mean values of hematological and biochemical indices in calves with respiratory troubles. The total white blood cell counts were higher in all diseased groups in comparison with the corresponding controls (*P* < 0.05). The mean values of serum total proteins and globulins were significantly increased only in calves with bronchopneumonia (*P* < 0.05). The serum activities of AST were higher in buffalo claves with emphysema and interstitial pulmonary syndrome in comparison with the corresponding control group (*P* < 0.05). Arterial blood gas analysis revealed increased partial tension of carbon dioxide (PCO_2_) in all diseased. In contrast, the mean values of partial tension of oxygen (PO_2_) were decreased in all diseased groups (*P* < 0.05).

**Table 2:** Laboratory findings in calves with respiratory troubles (*n* = 35)

	Buffalo calves			Cattle calves		
	Control (*n* = 6)	Emphysema (*n* = 8)	Interstitial pulmonary syndrome (*n* = 7)	Control (*n* = 6)	Bronchopneumonia (*n* = 12)	Pleurisy (*n* = 8)
RBCs (× 10^12^/L)	7.7 ± 0.2	7.8 ± 0.2	7.5 ± 0.3	7.8 ± 0.2	8.2 ± 0.3	8.5 ± 0.3
PCV (%)	30 ± 1	28 ± 0.9	28 ± 1	27 ± 1	28 ± 0.7	29 ± 0.9
Hemoglobin (g/L)	99 ± 2.2	98 ± 1.6	100 ± 0.9	96 ± 3	94 ± 3	98 ± 2.3
WBCs (× 10^9^/L)	6.3 ± 0.2	7.8 ± 0.3^**^	11.7 ± 0.5^**^	7.7 ± 0.4	11 ± 1^*^	12.3 ± 0.8^**^
Total proteins (g/L)	60 ± 0.7	59 ± 1.4	60 ± 0.8	58 ± 1.5	64 ± 2.3^*^	63 ± 2.4
Albumin (g/L)	30 ± 0.5	29 ± 1	29 ± 0.6	26 ± 0.9	25 ± 1.1	26 ± 1.3
Globulins (g/L)	30 ± 0.4	30 ± 1	31 ± 1	32 ± 1.2	39 ± 2.5^*^	37 ± 2.6
AST (U/L)	45 ± 4	66 ± 6^*^	71 ± 6^**^	58 ± 6	77 ± 5^*^	76 ± 7^*^
GGT (U/L)	19 ± 0.8	22 ± 1.6	20 ± 0.7	24 ± 1.3	24 ± 1.4	23 ± 1.2
Arterial blood gas analysis						
pH	7.35 ± 0.002	7.34 ± 0.006	7.34 ± 0.004	7.36 ± 0.01	7.33 ± 0.01	7.36 ± 0.01
PCO_2_ (mmHg)	50 ± 2.2	62 ± 1.5^**^	69 ± 1.3^**^	48 ± 5	60 ± 3^*^	57 ± 2.2^*^
PO_2_ (mmHg)	85 ± 5	58 ± 3.3^**^	55 ± 2.3^**^	88 ± 8	62 ± 3^**^	66 ± 4^*^
Bicarbonate (mmol/L)	27 ± 0.7	27 ± 1	26 ± 0.8	29 ± 1	30 ± 1.1	29 ± 1.6
BE (mmol/L)	1.3 ± 0.5	0.4 ± 0.9	0.8 ± 0.6	2.2 ± 0.5	−0.1 ± 0.8	1.5 ± 0.6

^*^or ^**^ Means within a row of the same species are significantly different (*P* < 0.05) or (*P* < 0.01) from values of the corresponding control animals

The association between the laboratory parameters and various respiratory troubles is summarized in Table 3 for 26 calves with ultrasound lung score ≥ 1 and clinical respiratory score ≥ 5. It was noticed that only WBCs and PCO_2_ were significantly (*P* = 0.006) and (*P* = 0.004), respectively, indicated respiratory troubles in weaned calves. However, weak correlations were observed between ULS and WBCs (*r* = 0.47, *P* < 0.1) and ULS and PCO_2_ (*r* = 0.36, *P* < 0.05) (Table 4).

**Table 3:** Association between laboratory measures and various respiratory troubles in 26 calves with ultrasound lung score ≥ 1 and clinical respiratory score ≥ 5

Variable	Emphysema (*n* = 5)	Interstitial pulmonary syndrome (*n* = 5)	Bronchopneumonia (*n* = 10)	Pleurisy (*n* = 6)	*P*-value
RBCs (× 10^12^/L)	7.7 ± 0.23	7.9 ± 0.33	8.4 ± 0.37	8.9 ± 0.32	0.153
PCV (%)	28 ± 0.9	30 ± 1	28 ± 0.8	29 ± 1	0.686
Hemoglobin (g/L)	99 ± 2	101 ± 1.2	97 ± 3	100 ± 2.6	0.772
WBCs (×10^9^/L)	7.5 ± 0.3	12.2 ± 0.4	11.1 ± 1.1	13.3 ± 0.6	0.006
Total proteins (g/L)	60 ± 1.3	61 ± 0.8	66 ± 2.6	63 ± 3.1	0.366
Albumin (g/L)	29 ± 1.5	29 ± 1.7	25 ± 1.2	26 ± 1.7	0.139
Globulins (g/L)	31 ± 0.9	32 ± 1	41 ± 2.5	37 ± 3.5	0.058
AST (U/L)	62 ± 7.7	72 ± 7	81 ± 5.5	83 ± 6.1	0.175
GGT (U/L)	21 ± 2.1	20 ± 1	23 ± 1.5	21 ± 1	0.578
Arterial blood gas analysis					
pH	7.34 ± 0.01	7.33 ± 0.01	7.32 ± 0.01	7.35 ± 0.01	0.299
PCO_2_ (mmHg)	64 ± 1	72 ± 2	62 ± 2	60 ± 3	0.004
PO_2_ (mmHg)	53 ± 3	52 ± 2	55 ± 5	60 ± 4	0.163
Bicarbonate (mmol/L)	27 ± 1.2	27 ± 1	30 ± 1.2	29 ± 1.3	0.322
BE (mmol/L)	0.8 ± 0.9	0.4 ± 1.4	−0.7 ± 1.6	1.5 ± 0.8	0.694

**Table 4:** Pearson correlation coefficients between haematological and biochemical variables and ultrasound lung score in calves with respiratory troubles (*n* = 35)

Variables	Ultrasound lung score
RBCs	*r* = 0.28^NS^
PCV	*r* = 0.26^NS^
Hb	*r* = − 0.05^NS^
WBCs	*r* = 0.47^**^
Total proteins	*r* = 0.28^NS^
Albumin	*r* = − 0.14^NS^
Globulins	*r* = 0.32^NS^
AST	*r* = 0.26^NS^
GGT	*r* = 0.21^NS^
pH	*r* = 0.04^NS^
PCO_2_	*r* = 0.36^*^
PO_2_	*r* = 0.15^NS^
Bicarbonate	*r* = 0.13^NS^
Base excess	*r* = − 0.11^NS^

^*NS*^ Not significant; ^**^ *P* < 0.01; ^*^ *P* < 0.05

### Therapy and outcome

Calves were treated with 8.7 mg/kg amoxicillin trihydrate, clavulanic acid (Synulox®) injected once daily into the muscles of the neck or hindquarters. Bromhexine Hydrochloride (Bisolvon) in a dose 0.5 mg/kg per os. 0.5 mg/kg meloxicam (metacam) intravenous injection. The clinical signs including CRS and appetite were improved in most diseased calves, therefore; they were discharged from the hospital one or 3 days after beginning of treatment and the importance of completing the therapeutic course was emphasized to the owner and referring veterinarian.

**Fig. 8:**
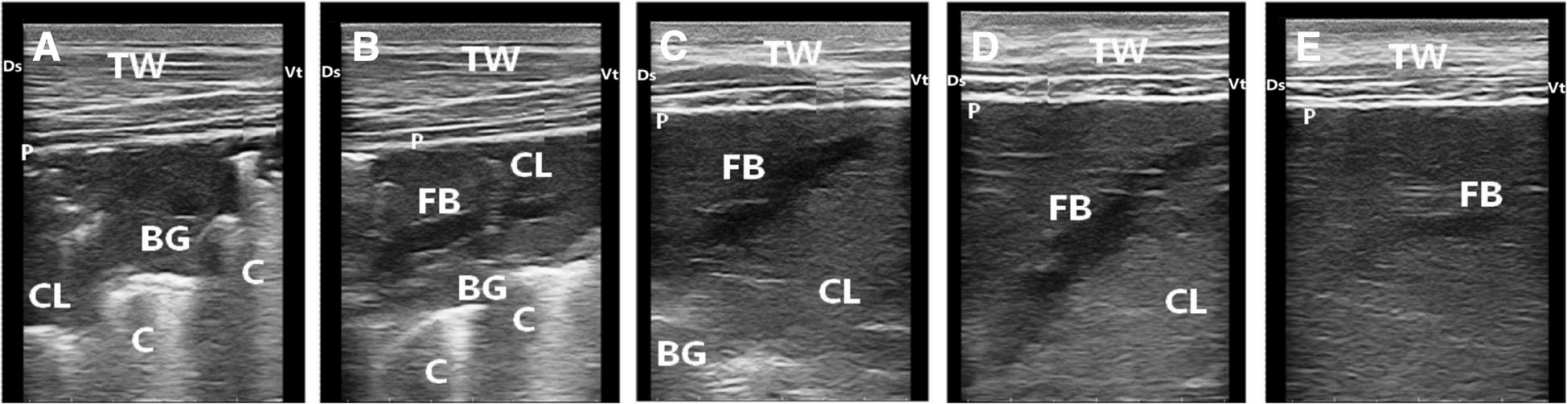
Ultrasonograms of a calf with severe bronchopneumonia show the development of pulmonary lesions during the course of therapy. Image (**a**), at admission of the calf, reveals consolidated cranial lobe (CL) with hyperechoic bronchoaerogram (BG). Image (**b**) for the same calf after 5 days of therapy shows consolidated cranial lobe (CL), bronchoaerogram (BG) and fluid bronchogram (FB). Image (**c**), after 10 days of therapy, exhibits consolidated lung (CL) and few bronchoaerograms (BG). Image (**d**), after 20 days of therapy, reveals improvement of pulmonary lesions but lung consolidation (CL) and fluid bronchogram (FB) are still present. Image (**e**), after 30 days of therapy, shows an obvious improvement in the pulmonary tissue in comparison with previous images but a remnant of fluid bronchogram (FB) is still present. TW, Thoracic wall; P, Pleura; Ds, Dorsal; Vt, Ventral

Ten cattle calves with bronchopneumonia were severely diseased and they kept in the hospital for a month and examined clinically and ultrasonographically for assessment of therapy. After 3 days from the beginning of treatment, coughing and nasal discharges were much reduced. The clinical respiratory score was improved after 5 days (Table 5). Few crackle sounds were heard over lung lesion. Crackles were not consistently identified in 6 calves despite the presence of lung hepatization noted during ultrasonographic examination. Follow-up ultrasound examination of calves revealed gradual improvement and tissue healing (Fig. 8). One incurable case was euthanased and sent for necropsy examination, which revealed gross lung pathology and exudation.

**Table 5:** Pre- and post-therapeutic variations of CRS, ULS and laboratory indices in calves with bronchopneumonia (*n* = 10)

	Control (*n* = 6)	Pre- and post-therapeutic days				
		At admission (0)	5	10	20	30
CRS	1.7 ± 0.2	5.4 ± 0.2^a^	3.8 ± 0.2^b^	2.6 ± 0.2^c^	1.8 ± 0.2^d^	1.5 ± 0.2^d^
ULS	0.0 ± 0.0	3.4 ± 0.3^a^	3.2 ± 0.2^ab^	2.9 ± 0.3^abc^	2.6 ± 0.2^bc^	2.3 ± 0.1^c^
RBCs (×10^12^/L)	7.8 ± 0.2	8.4 ± 0.4	8.1 ± 0.3	7.8 ± 0.2	8.2 ± 0.1	8.0 ± 0.1
PCV (%)	27 ± 1	28 ± 0.8	29 ± 0.6	29 ± 0.7	28 ± 0.3	29 ± 0.4
Hemoglobin (g/L)	96 ± 3	97 ± 3	99 ± 2	98 ± 2	102 ± 1	99 ± 1
WBCs (×10^9^/L)	7.7 ± 0.4	11.7 ± 1^a^	10.5 ± 0.9^ab^	9.3 ± 0.4^b^	8.8 ± 0.2^b^	9 ± 0.2^b^
Total proteins (g/L)	58 ± 1.5	66 ± 3	65 ± 2	63 ± 2	62 ± 1	63 ± 1
Albumin (g/L)	26 ± 0.9	25 ± 1	24 ± 0.9	25 ± 0.7	26 ± 1.1	27 ± 0.6
Globulins (g/L)	32 ± 1.2	41 ± 2	41 ± 2	38 ± 2	36 ± 1	36 ± 1
AST (U/L)	62 ± 6	81 ± 5^a^	83 ± 3^a^	81 ± 3^a^	78 ± 3^ab^	69 ± 3^b^
GGT (U/L)	24 ± 1.3	23 ± 1.5	21 ± 0.9	20 ± 0.5	22 ± 0.5	21 ± 0.5
Arterial blood gas analysis						
pH	7.36 ± 0.01	7.33 ± 0.01	7.32 ± 0.01	7.31 ± 0.02	7.32 ± 0.01	7.32 ± 0.01
PCO_2_ (mmHg)	48 ± 2	56 ± 3^a^	54 ± 3^ab^	51 ± 1^ab^	50 ± 2^ab^	49 ± 1^b^
PO_2_ (mmHg)	88 ± 2	58 ± 3^b^	66 ± 3^ab^	69 ± 2^ab^	73 ± 2^ab^	75 ± 3^a^
Bicarbonate (mmol/L)	29 ± 1	29 ± 0.8	29 ± 0.7	28 ± 0.6	27 ± 0.6	27 ± 0.5
BE (mmol/L)	2.2 ± 0.5	−0.7 ± 1.6	0.9 ± 0.3	0.6 ± 0.3	0.8 ± 0.1	0.6 ± 0.2

*CRS* Clinical respiratory score, *ULS* ultrasound lung score

^a,b,c,d^ values of different superscript letters in the same raw differs significantly (*P* < 0.05)

## Discussion

Clinical findings of deteriorated body condition, coughing, and variable degrees of nasal discharges, dyspnea and respiratory noises in all diseased groups were supported in a previous study of bovine pulmonary diseases [15]. In present study, most of diseased groups showed increased body temperature except those with emphysema, indicating acute stage of respiratory disease in the pyretic groups. In a previous report [16], the authors indicated fever > 39.7 °C as the most important selection criterion for antibiotic therapy in growing cattle with acute respiratory illness.

Radiography, computed tomography (CT), and ultrasonography are non-invasive clinical tools for diagnosing respiratory troubles antemortem. Radiology had a good sensitivity and poor specificity [17]. In a previous study [18], a high correlation was found between CT and postmortem levels of lung consolidations in young dairy calves. However, radiography and CT are not practical for diagnosing respiratory diseases in large numbers of calves in a farm setting because of physical equipment constraints, expenses, anaesthetic requirements, and the hazards of exposure to radiation [19]. In contrast, thoracic ultrasonography can be carried out calf-side using readily transportable machines without the fear of exposure to radiation, as well as no special health and safety procedures or restrictions are required.

Ultrasonographic examination of normal air-filled pulmonary tissue appeared as a hyperechoic line representing the visceral pleura due to total reflection of ultrasound waves [20]. Furthermore, presence of reverberation artifacts may be attributed to block the progression of the ultrasound waves by the air contained in the lung tissue [14].

Thoracic ultrasonography in buffalo calves with pulmonary emphysema revealed narrow comet-tail artifacts extended from the pleural surface and downward. In cattle, Flöck [21] also reported the presence of comet-tail artifacts in the presence of pulmonary emphysema. In addition, buffalo calves with interstitial pulmonary syndrome showed broad ultrasound lung comets that appeared on the monitor as white images, indicating diffuse pulmonary lesions. According to the authors’ knowledge, no studies in animals reporting the ultrasound description of interstitial pulmonary syndrome are available. Therefore, this report described for the first time the ultrasound imaging of interstitial pulmonary syndrome in calves. Diffuse parenchymal lung disease must be considered if the multiple comet-tail artifacts are distributed over the entire lung surface [22] In human medicine, Lichtenstein and Mezière [23] attributed these ultrasound comets to the thickening of subpleural interlobular septa, which would cause fragmentation of the pleural specular reflector at the points of greatest impedance. In the present study, this variation between the two types of comets of pulmonary emphysema and interstitial syndrome may be due to the differences in the pathogenesis and lesions of both respiratory diseases. Furthermore, this ultrasound variation can be used as an important item for diagnosis and differential diagnosis of the two diseased conditions, especially the physical examinations of animals in both groups revealed no obvious sharp differences.

Ultrasonographic examinations of cattle calves with bronchopneumonia revealed presence of pulmonary consolidations, where the lung tissues appeared hypoechoic and their echo texture may look like liver parenchyma containing bronchoaerograms and/or brochograms in this region. As reported elsewhere [19], bronchoaerograms are small bronchi filled with air, which makes them appear hyperechoic, while bornchograms are anechoic tubular structures, representing fluid filled bronchi. In the current study, bronchograms resembled the blood vessels in the echogenicity. Therefore, Doppler ultrasonography was used to distinguish between blood vessels and bronchograms. Bronchograms appeared lacking the blood flow. Thoracic ultrasonography of calves with pleurisy showed varied images ranged from thickening and irregularity to fragmentation with pleural effusion of pleurae, indicating the degree and severity of illness. As mentioned before [24], ultrasonography allows evaluation of pleura and permits pleural effusion to be visualized and quantifies the nature and extent of the effusion.

For evaluation of prognosis, periodical follow-up ultrasonography of bronchopneumonic calves during therapy showed gradual improvement of ultrasound lung scores, indicating healing of the pulmonary tissues. In contrast, clinical respiratory scores were weakly correlated with ultrasound lung scores; this may be explained as the clinical signs of respiratory problems diminished or subsided while the pulmonary lesions were still existed. It may be a misleading and a false decision about the case prognosis may be taken by the bovine veterinarians if they depend only on the physical examinations and consequently the disease relapse may occur. Therefore, it may be advisable to use thoracic ultrasonography as a follow-up tool for monitoring the efficacy of therapy. As mentioned elsewhere [25], the type, severity, and extent of pulmonary diseases cannot always be determined by clinical examination alone and this may lead to misinterpretation of respiratory symptoms and ineffective therapy.

In the present study, hematological analysis revealed increased white blood cell counts in all diseased groups. In addition, white blood cell counts were associated with various respiratory troubles, indicating active inflammatory reactions in the lung tissues. However, many diseases may exhibit such increases as a consequence of inflammatory processes like in respiratory diseases [26]. In calves with bronchopneumonia, increased concentrations of serum total proteins and globulins could be attributed to the acute phase response as a result of pulmonary infection. As mentioned before [27], the demand for amino acids for synthesis of the positive acute phase proteins is markedly increased during the acute phase reaction. In the present study, AST activities were significantly increased, which may be due to increased respiratory rates and consequently increased efforts of intercostal muscles with prolonged pulmonary diseases. This is in agreement with a previous report [28]. However, AST is not a specific parameter to a particular organ, as it originates from many tissues but muscles and liver are considered to be its major sources [29]. In the current study, arterial blood gas analysis showed increased PCO_2_ and decreased PO_2_ in diseased calves, indicating impaired lung function. However, PCO_2_ and PO_2_ failed to distinguish among the various respiratory diseases. As mentioned elsewhere [30], the authors reported that disturbed partial tensions of oxygen and carbon dioxide may develop as a result of decreased effective alveolar ventilation. In contrast, Tharwat and Oikawa [31] reported no changes in arterial blood gases in cattle with respiratory disorders. Such difference may be attributed to the variations in the degree and severity of illness. In this study, it was noticed that only white blood cells and PCO_2_ were associated with respiratory troubles. However, weak correlations were observed between these two variables and ultrasound lung scores, indicating these parameters may be not specific for pulmonary lesions.

## Conclusions

Thoracic ultrasonography is a helpful tool for diagnosis of various respiratory troubles and assessment the grade and severity of pulmonary lesions. The clinical signs of respiratory disease may subside and the respiratory scores may improve in a time earlier than the pulmonary lesions disappear. Therefore, the thoracic ultrasonography can be used as a follow-up tool for evaluating the prognosis of respiratory troubles and monitoring the efficacy of respiratory therapies.

## Abbreviations

AST: Aspartate aminotransferase
BE: Base excess
CRS: Clinical respiratory score
CT: Computed tomography
GGT: γ-glutamyl transpeptidase
ICS: Intercostal space
PCO_2_: Partial tension of oxegen
PO_2_: Partial tension of oxygen
RBCs: Red blood cells
ULS: Ultrasound lung score
WBCs: White blood cells
